# Supplementary material for: IGF1R-phosphorylated PYCR1 facilitates ELK4 transcriptional activity and sustains tumor growth under hypoxia
Source: Nat Commun. 2023 Sep 30;14:6117. doi: 10.1038/s41467-023-41658-z (PMC10542766; doi:10.1038/s41467-023-41658-z)
Supplement: Supplementary file 1 — Supplementary Information [file 41467_2023_41658_MOESM1_ESM.pdf]

Supplementary information

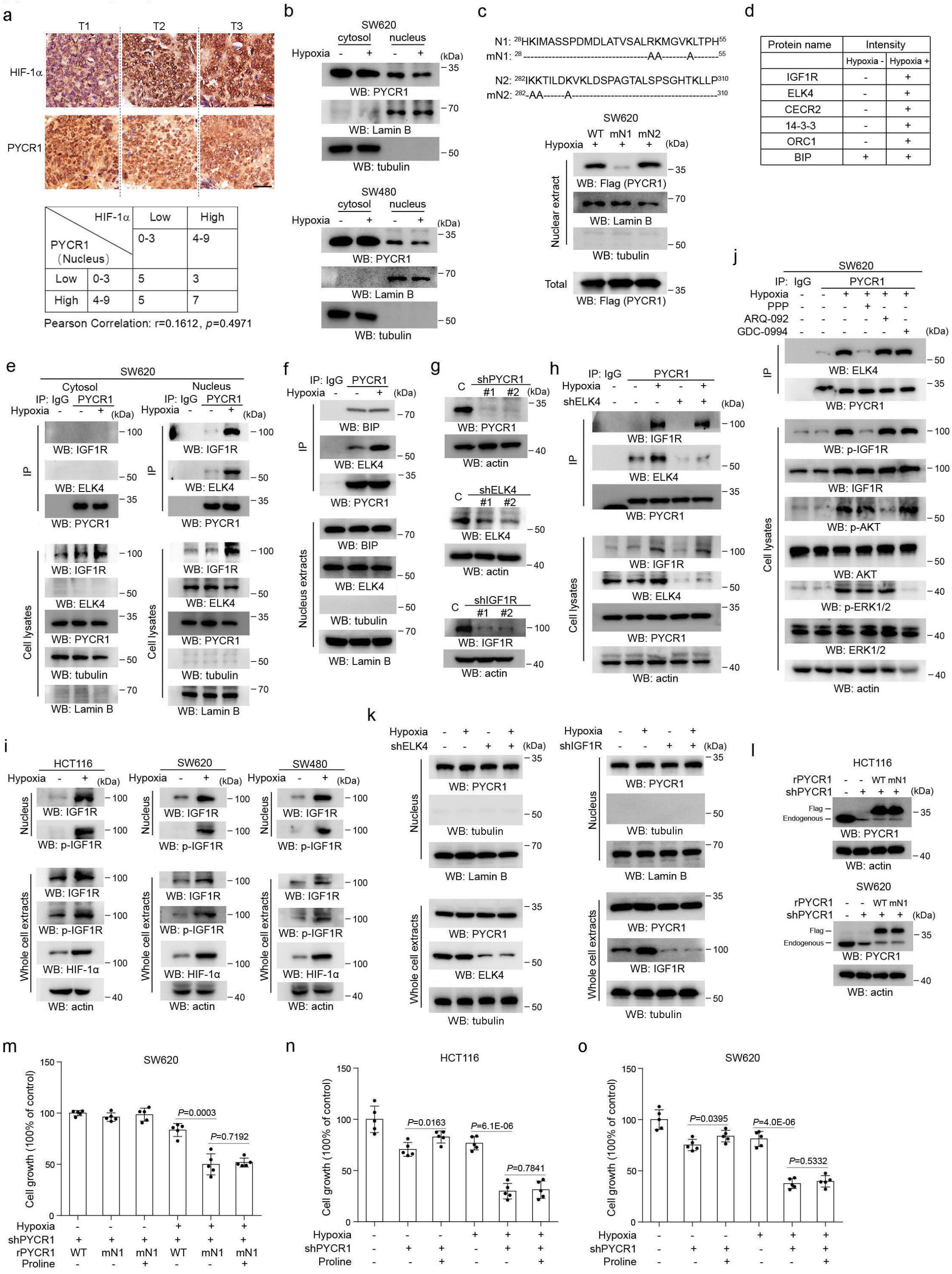

Supplementary Fig. 1. The nuclear localization of PYCR1 is critical for tumor cell growth under hypoxia. In b, c and e-l, immunoblotting and immunoprecipitation analysis were performed using the indicated antibodies. In m, n and o, the values are presented as mean ± s.d. (n = 5 independent experiments); statistical analysis was performed using the two-tailed Student's t-test. Source data are provided as Source Data

uncropped western blots and Source Data Supplementary Fig.1. (a) Immunohistochemical staining with anti-HIF-1α and anti-PYCR1 antibodies was performed on human colorectal cancer specimens and representative images were shown. Scale bars: 50 μm (magnification: ×400). Semiquantitative intensity scoring and Pearson correlation analysis of 20 tumor sections indicated the correlation of nuclear PYCR1 with HIF-1α (r=0.1612, p=0.4971). (b) SW620 or SW480 cells were cultured under normoxia or hypoxia for 12 h and cytosolic and nuclear extracts were collected. (c) SW620 cells expressing indicated Flag-tagged PYCR1 were cultured under hypoxia for 12 h, and the nuclear extracts were collected. (d) The table shows mass spectrometry-identified proteins that were specifically associated with PYCR1 under hypoxia. (e) SW620 cells were cultured under normoxia or hypoxia for 12 h. The cytosolic (left panel) or nuclear extracts (right panel) were collected. (f) HCT116 cells were cultured under normoxia or hypoxia for 12 h. (g) HCT116 cells were transfected with the indicated shRNA and the efficiency for protein knockdown was examined. (h) HCT116 cells transfected with ELK4 shRNA were cultured under normoxia or hypoxia for 12 h. (i) The indicated cell lines were cultured under normoxia or hypoxia for 12 h, the nuclear extracts were collected. (j) SW620 cells were pretreated with PPP (1 μM), ARQ-092 (2 μM) and GDC-0994 (10 μM) for 1 h before being cultured under hypoxia for 12 h. (k) HCT116 cells transfected with ELK4 shRNA or IGF1R shRNA were cultured under normoxia or hypoxia for 12 h, the nuclear extracts were collected. (l) HCT116 and SW620 cells with depletion of PYCR1 were reconstituted with expression of Flag-tagged shRNA resistant PYCR1. (m) SW620 cells with depletion of PYCR1 and reconstituted with expression of indicated Flag-rPYCR1 were treated with or without exogenous proline (3 mM). Cells were cultured under normoxia or hypoxia for 48 h. Cellular viability was examined by CCK-8 assay. (n, o) HCT116 (n) or SW620 (o) cells depleted of PYCR1were treated with or without exogenous proline (3 mM). Cells were cultured under normoxia or hypoxia for 48 h. Cellular viability was examined by CCK-8 assay.

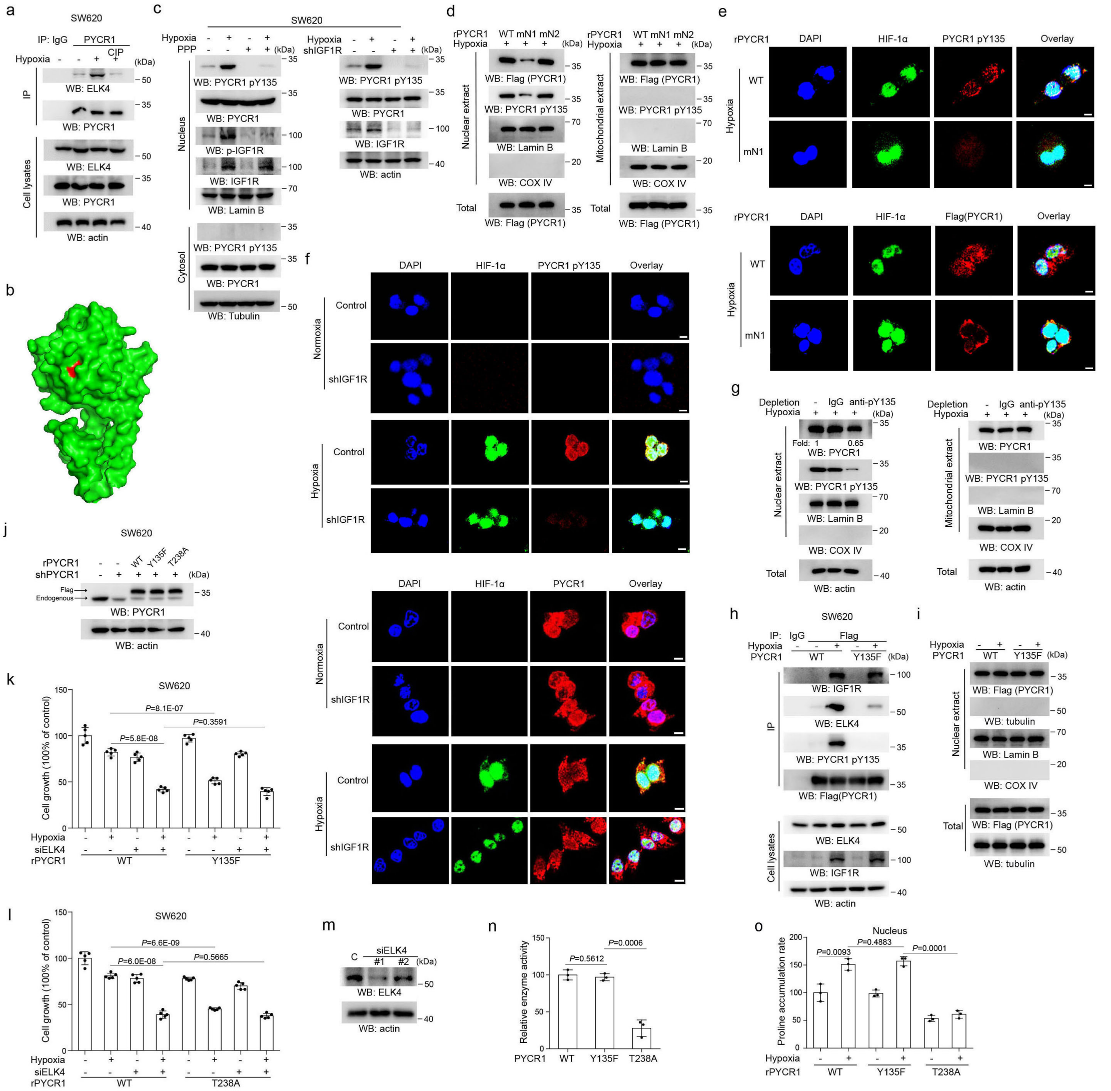

Supplementary Fig. 2. IGF1R phosphorylates PYCR1 and promotes PYCR1-ELK4 interaction. In a, c, d, g-j and m, immunoprecipitation and immunoblotting analysis were performed using the indicated antibodies. In e and f, immunofluorescence analysis was performed using indicated antibodies. In k, l, n and o, the values are presented as mean ± s.d., statistical analysis was performed using the two-tailed Student's *t*-test. Source data are provided as Source Data uncropped western blots and Source Data Supplementary Fig.2. (a) SW620 cells were cultured under hypoxia for 12 h. The immunoprecipitates were treated with CIP (10 units). (b) Human PYCR1 structure shows Tyr-135 (Red marked) is a protein surface-locating residue (PDB ID: 2GRA). (c) SW620 cells pretreated with or without PPP (1 μM) for 1 h (left panel). SW620 cells transfected with or without IGF1R siRNA (right panel). Cells were cultured under normoxia or hypoxia for 12 h and cytosolic or nuclear extracts were

collected. (d) SW620 cells expressing indicated Flag-PYCR1s were cultured under normoxia or hypoxia for 12 h. (e) HCT116 cells expressing indicated Flag-PYCR1s were cultured under hypoxia for 12 h. (f) HCT116 cells transfected with or without IGF1R shRNA were cultured under normoxia or hypoxia for 12 h. (g) HCT116 cells were cultured under hypoxia for 12 h. The nuclear (left panel) or mitochondrial (right panel) extracts collected were incubated with IgG or anti-PYCR1 pY135 antibody. (h, i) SW620 cells expressing indicated Flag-PYCR1s were cultured under normoxia or hypoxia for 12 h. Immunoprecipitation analysis was performed (h); immunoblotting analysis was performed as indicated (i). (j) SW620 cells with depletion of endogenous PYCR1 were reconstitutively expressed with indicated Flag-rPYCR1s. (k, l) SW620 cells with reconstituted expression of WT rPYCR1 and rPYCR1 Y135F (k) or rPYCR1 T238A (l) were transfected with or without ELK4 siRNA. Cells were cultured under normoxia or hypoxia for 48 h. Cellular viability was examined by CCK-8 assay ($n = 5$ independent experiments). (m) HCT116 cells were transfected with or without ELK4 siRNA. (n) WT and mutant His-PYCR1 were purified and the enzymatic activity was measured ($n = 3$ independent experiments). (o) HCT116 cells expressing indicated Flag-rPYCR1s were cultured under normoxia or hypoxia for 24 h. The proline level in nuclear fraction was measured ($n = 3$ independent experiments).

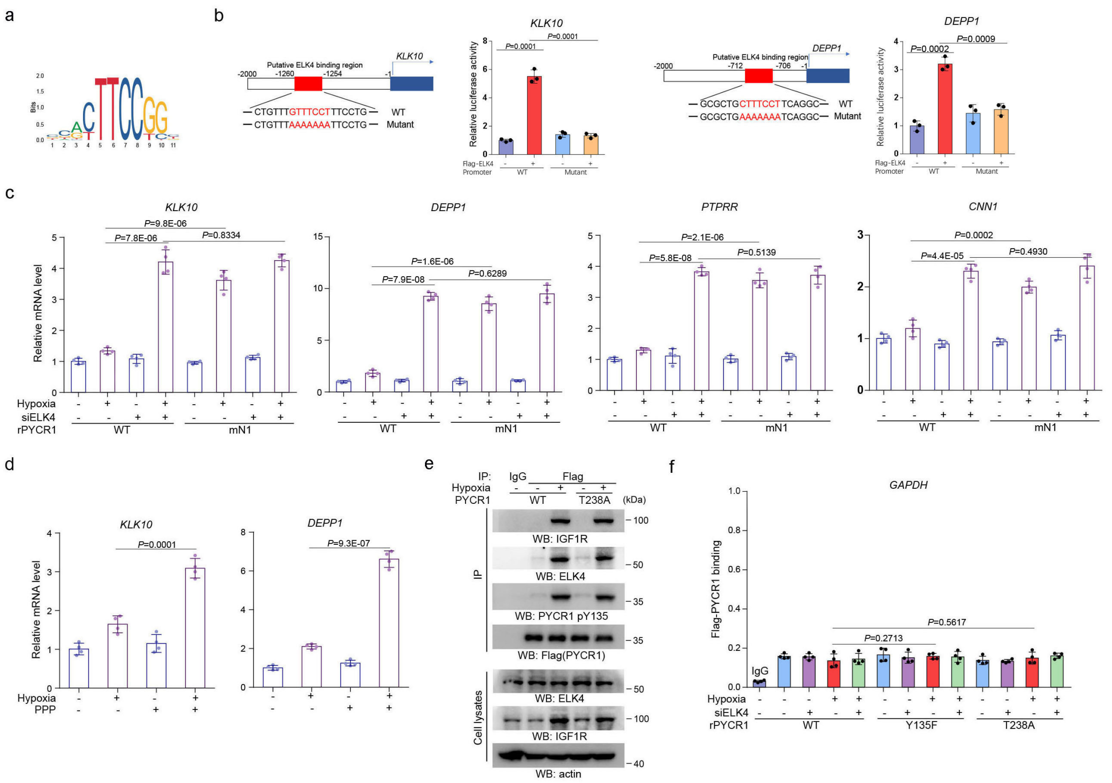

Supplementary Fig. 3. PYCR1 facilitates ELK4-mediated transcriptional repression for growth maintenance. In b-d and f, the values are presented as mean ± s.d. (*n* = 3 independent experiments for b; *n* = 4 independent experiments for c, d and f); statistical analysis was performed using the two-tailed Student's *t*-test. Source data are provided as Source Data uncropped western blots and Source Data Supplementary Fig.3. (a) The diagram showing the ELK4-binding consensus sequence noted as C/TTTCC. (b) Simplified schematic showing the response element constructs with putative ELK4-binding consensus sequence in the *KLK10* or *DEPP1* promoter (1st and 3rd panels). HEK293T cells were overexpressed with or without the ELK4 expression vector. Luciferase reporter assays were performed (2nd and 4th panels). (c) HCT116 cells expressing indicated Flag-rPYCR1s were transfected with or without ELK4 siRNA. Cells were cultured under hypoxia for 12 h. mRNA levels of indicated ELK4 target genes were analyzed by real-time PCR. (d) HCT116 cells pretreated with or without PPP (1 μM) for 1 h before cultured under hypoxia for 24 h. mRNA levels of indicated ELK4 target genes were analyzed by real-time PCR. (e) HCT116 cells expressing indicated Flag-rPYCR1s were cultured under normoxia or hypoxia for 12 h. Immunoprecipitation were performed using the indicated antibodies. (f) Cells

expressing the indicated Flag-rPYCR1 were cultured under hypoxia for 12 h. ChIP analysis were performed using the indicated antibody. The primers covering the promoter regions of the indicated gene were utilized for real-time PCR analysis. The y axis shows the value normalized to the input.

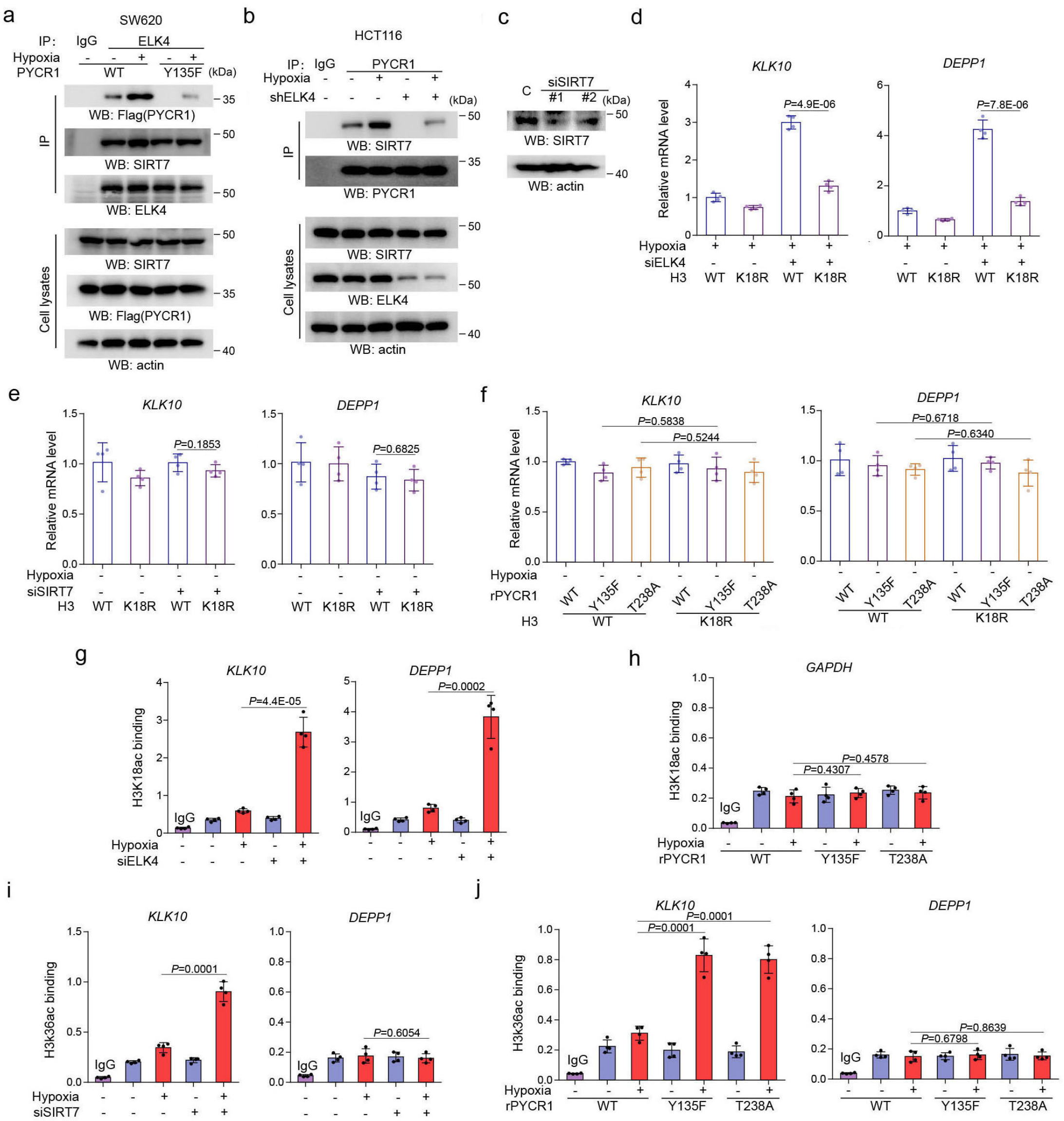

Supplementary Fig. 4. PYCR1 facilitates Sirt7-mediated H3K18 deacetylation. In a-c, immunoblotting or immunoprecipitation were performed using the indicated antibodies. In d-f, mRNA levels of indicated ELK4 target genes were analyzed by real-time PCR. In g-j, ChIP analysis were performed with indicated antibodies, the primers covering the ELK4-binding sites at promoter regions of the indicated genes were utilized for real-time PCR analysis. The y axis shows the value normalized to the input. In d-j, the values are presented as mean ± s.d. (n = 4 independent experiments); statistical analysis was performed using the two-tailed Student's t-test. Source data are provided as Source Data uncropped western blots and Source Data Supplementary Fig.4. (a) SW620 cells expressing indicated Flag-PYCR1s were cultured under normoxia or hypoxia for 12 h. (b) HCT116 cells transfected with or without ELK4

siRNA were cultured under normoxia or hypoxia for 12 h. (c) HCT116 cells were transfected with SIRT7 siRNA and the efficiency was examined. (d) HCT116 cells expressing WT Histone H3 or Histone H3 K18R were transfected with or without ELK4 siRNA. Cells were cultured under hypoxia for 12 h. mRNA levels of indicated ELK4 target genes were analyzed by real-time PCR. (e, f) HCT116 cells expressing WT Histone H3 or Histone H3 K18R were transfected with or without SIRT7 siRNA (e) or expressed with indicated Flag-rPYCR1s (f). Cells were cultured under normoxia for 12 h. mRNA levels of indicated ELK4 target genes were analyzed by real-time PCR. (g, h) HCT116 cells transfected with or without ELK4 siRNA (g) or expressing indicated Flag-rPYCR1s (h) were cultured under normoxia or hypoxia for 12 h. (i, j) HCT116 cells transfected with or without SIRT7 siRNA (i) or expressing indicated Flag-rPYCR1s (j) were cultured under normoxia or hypoxia for 12 h.

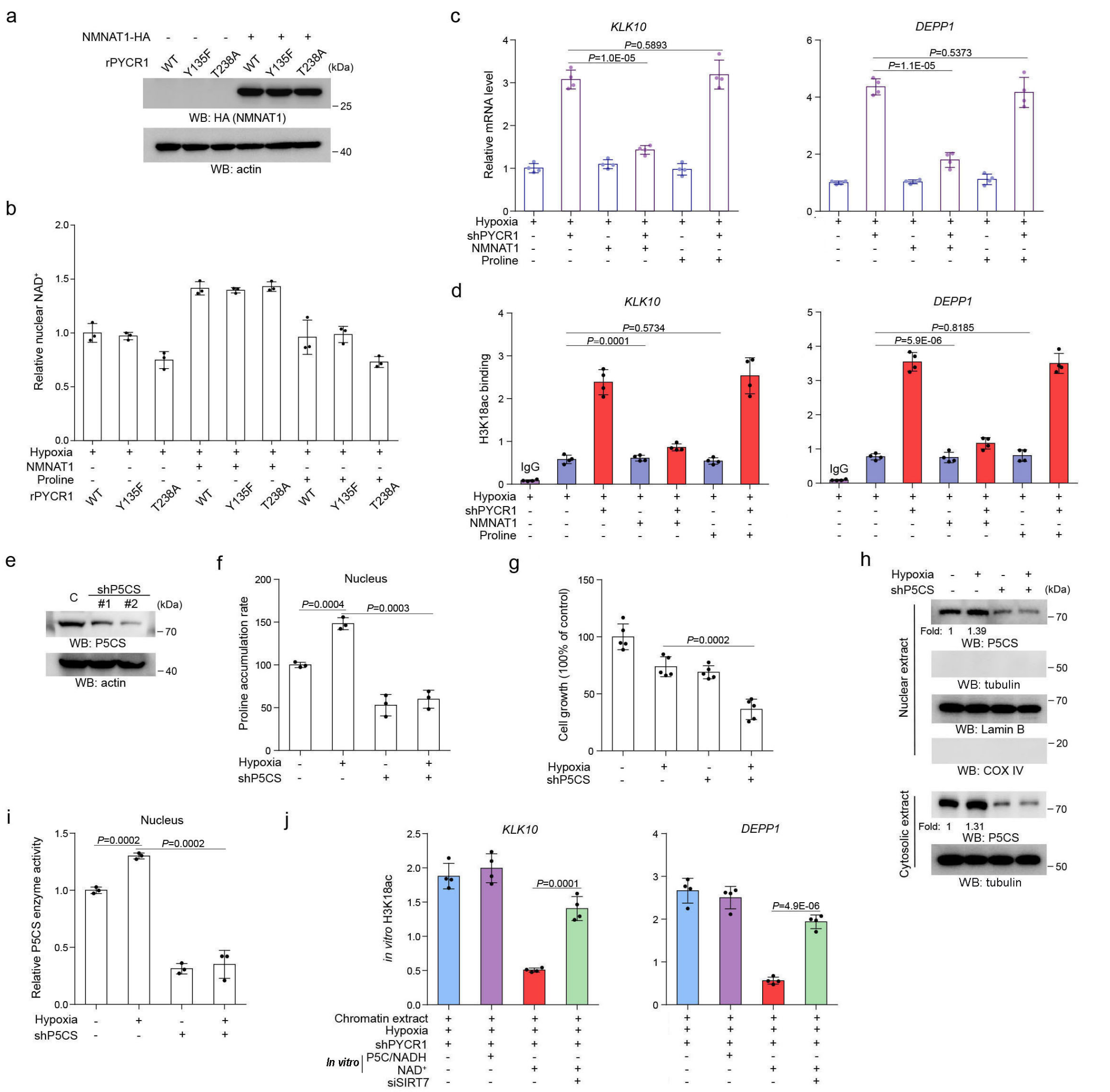

Supplementary Fig. 5. PYCR1-catalyzed NAD+ production is involved in transcriptional regulation. In b, c, d, f, g, i and j, the values are presented as mean ± s.d.; statistical analysis was performed using the two-tailed Student's *t*-test. Source data are provided as Source Data uncropped western blots and Source Data Supplementary Fig.5. (a, b) HCT116 cells expressing the indicated Flag-rPYCR1 were overexpressed with or without NMNAT1-HA. (a) The immunoblotting analysis was performed using the indicated antibodies. (b) The cells were supplemented with or without the indicated metabolites, and the NAD+ content in nuclear fraction was measured using the NAD+ assay kit (n = 3 independent experiments). (c, d) HCT116 cells were transfected with or without PYCR1 shRNA. Cells simultaneously overexpressed with or without NMNAT1 were supplemented with the metabolites as indicated. Cells were cultured under hypoxia for 24 h (c) or 12 h (d). Real-time PCR (c) or ChIP (d) analysis was

performed (*n* = 4 independent experiments). (e) HCT116 cells were transfected with or without P5CS shRNA. The immunoblotting analysis of the knockdown efficiency was performed using indicated antibodies. (f) HCT116 cells transfected with or without P5CS shRNA were cultured under normoxia or hypoxia for 24 h. The proline level in nuclear fraction was measured (*n* = 3 independent experiments). (g) HCT116 cells with or without P5CS knockdown were cultured under normoxia or hypoxia for 48 h. Cellular viability was examined by CCK-8 assay (*n* = 5 independent experiments). (h) HCT116 cells transfected with P5CS shRNA were cultured under normoxia or hypoxia for 24 h. The immunoblotting analysis was performed using indicated antibodies. (i) HCT116 cells were transfected with or without P5CS shRNA. P5CS enzymatic activity was examined (*n* = 3 independent experiments). (j) HCT116 cells depleted of PYCR1 were transfected with or without Sirt7 siRNA. Cells were cultured under hypoxia for 12 h. Chromatin extracts were collected and mixed with or without the indicated metabolites for 30 min, and then chromatin extracts were fixed and used for ChIP analysis (*n* = 4 independent experiments).

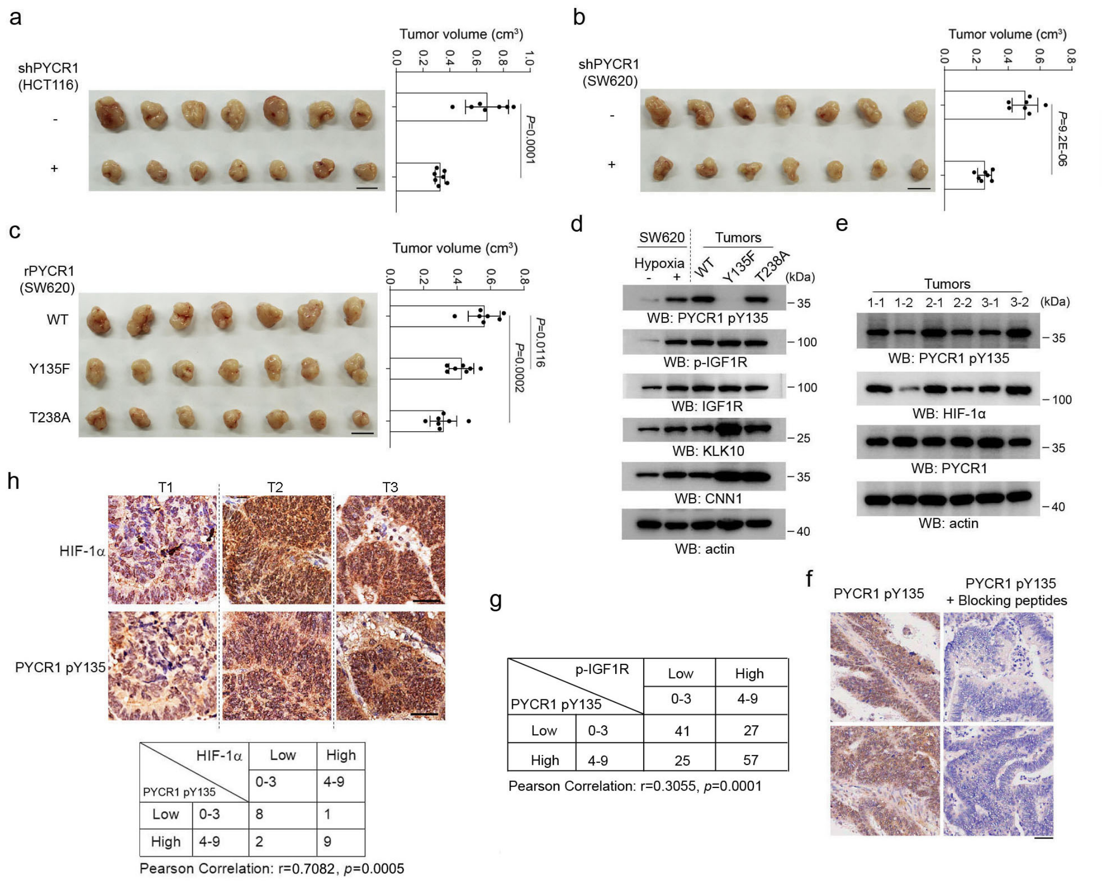

Supplementary Fig. 6. PYCR1 Y135 phosphorylation facilitates tumor development in colorectal cancer. In a, b and c, representative tumor xenografts were shown (left panel). Scale bars: 1 cm. Tumor volumes were measured by using length "a" and width "b" and calculated using the following equation: $V = ab^2/2$. Data are represented as mean ± s.d. ($n = 7$, right panel). Statistical analysis was performed using the two-tailed Student's *t*-test. Source data are provided as Source Data uncropped western blots and Source Data Supplementary Fig.6. (a) A total of 3×10^6 HCT116 cells transfected with or without PYCR1 shRNA were subcutaneously injected into the athymic nude mice. (b) A total of 5×10^6 SW620 cells transfected with or without PYCR1 shRNA were subcutaneously injected into the athymic nude mice. (c) A total of 5×10^6 SW620 cells expressing the indicated Flag-rPYCR1 were subcutaneously injected into the athymic nude mice. (d) Lysates collected from SW620 cells cultured under hypoxia and from tumour tissues were subjected to immunoblotting analysis. (e) Tumor tissues were isolated from two distinct regions in three mice tumors (Labeled as 1-1, 1-2, 2-1, 2-2, 3-1 and 3-2) derived from SW620 cells expressing WT rPYCR1, then each part was subjected to immunoblotting analysis. (f) The PYCR1 pY135 antibody

specificity was validated using IHC analysis with specific blocking PYCR1 pY135 peptides. Scale bars: 100 μm. (g) Semiquantitative scoring and Pearson correlation analysis indicating the correlation between PYCR1 pY135 and p-IGF1R (r=0.3055, p=0.0001) (n = 150 human colorectal tumour specimens). (h) Immunohistochemical staining with anti-HIF-1α and anti-PYCR1 pY135 antibodies was performed on human colorectal cancer specimens and representative images were shown. Scale bars: 50 μm (magnification: ×400). Semiquantitative intensity scoring and Pearson correlation analysis of 20 tumor sections indicated the correlation of PYCR1 pY135 with HIF-1α expression (r=0.7082, p=0.0005).
