# Supplementary material for: IGF1R-phosphorylated PYCR1 facilitates ELK4 transcriptional activity and sustains tumor growth under hypoxia
Source: Nat Commun. 2023 Sep 30;14:6117. doi: 10.1038/s41467-023-41658-z (PMC10542766; doi:10.1038/s41467-023-41658-z)
Supplement: Supplementary file 2 — Description of Additional Supplementary Files [file 41467_2023_41658_MOESM2_ESM.pdf]

Description of Additional Supplementary Files

Supplementary Data 1. The differential expression genes (DEGs) between WT rPYCR1- and rPYCR1 Y135F-expressing HCT116 cells under hypoxia. Differential expression analysis was performed using the edgeR. DEGs with $|log2FC| \geq 1$ and P-value ≤ 0.05 were considered to be significantly different expressed genes. A1 represents WT rPYCR1, A4 represents rPYCR1 Y135F.

Supplementary Data 2. The genes whose promoter region maps ELK4-binding consensus sequence (CCGGA or C/TTTCC) in Supplementary Data file 1.
