# Supplementary material for: IGF1R-phosphorylated PYCR1 facilitates ELK4 transcriptional activity and sustains tumor growth under hypoxia
Source: Nat Commun. 2023 Sep 30;14:6117. doi: 10.1038/s41467-023-41658-z (PMC10542766; doi:10.1038/s41467-023-41658-z)
Supplement: Supplementary file 5 — Reporting Summary [file 41467_2023_41658_MOESM5_ESM.pdf]

nature portfolio

Corresponding author(s): Tao Chen

Last updated by author(s): Aug 12, 2023

Reporting Summary

Nature Portfolio wishes to improve the reproducibility of the work that we publish. This form provides structure for consistency and transparency in reporting. For further information on Nature Portfolio policies, see our Editorial Policies and the Editorial Policy Checklist.

Statistics

For all statistical analyses, confirm that the following items are present in the figure legend, table legend, main text, or Methods section.

n/a	Confirmed	
☐	☒	The exact sample size (*n*) for each experimental group/condition, given as a discrete number and unit of measurement
☐	☒	A statement on whether measurements were taken from distinct samples or whether the same sample was measured repeatedly
☐	☒	The statistical test(s) used AND whether they are one- or two-sided<br>*Only common tests should be described solely by name; describe more complex techniques in the Methods section.*
☐	☒	A description of all covariates tested
☐	☒	A description of any assumptions or corrections, such as tests of normality and adjustment for multiple comparisons
☐	☒	A full description of the statistical parameters including central tendency (e.g. means) or other basic estimates (e.g. regression coefficient) AND variation (e.g. standard deviation) or associated estimates of uncertainty (e.g. confidence intervals)
☐	☒	For null hypothesis testing, the test statistic (e.g. *F*, *t*, *r*) with confidence intervals, effect sizes, degrees of freedom and *P* value noted<br>*Give P values as exact values whenever suitable.*
☒	☐	For Bayesian analysis, information on the choice of priors and Markov chain Monte Carlo settings
☒	☐	For hierarchical and complex designs, identification of the appropriate level for tests and full reporting of outcomes
☒	☐	Estimates of effect sizes (e.g. Cohen's *d*, Pearson's *r*), indicating how they were calculated

Our web collection on statistics for biologists contains articles on many of the points above.

Software and code

Policy information about availability of computer code

Data collection	For data collection we used Excel (V2019, Microsoft, Redmont, WA).
Data analysis	Data analyses were performed utilizing SPSS Statistics 20 (SPSS, Chicago, IL, USA) for the graphic statistics.

For manuscripts utilizing custom algorithms or software that are central to the research but not yet described in published literature, software must be made available to editors and reviewers. We strongly encourage code deposition in a community repository (e.g. GitHub). See the Nature Portfolio guidelines for submitting code & software for further information.

Data

Policy information about availability of data

All manuscripts must include a data availability statement. This statement should provide the following information, where applicable:

- Accession codes, unique identifiers, or web links for publicly available datasets
- A description of any restrictions on data availability
- For clinical datasets or third party data, please ensure that the statement adheres to our policy

All relevant data are available from the authors.

Human research participants

Policy information about studies involving human research participants and Sex and Gender in Research.

Reporting on sex and gender	No reporting on sex and gender.
Population characteristics	Human tumour samples and their paired noncancerous matched tissues were obtained from Colorectal cancer patients undergoing surgery. Survival time was calculated from the date of surgery to the date of death or last follow-up.
Recruitment	Human tumour samples were obtained from a series of 150 CRC patients with surgery. Patients with radiotherapy or chemotherapy treatment before surgery were excluded.
Ethics oversight	The study was approved by Institutional Review Board (IRB) of the Ethics Committee of Shanghai East Hospital, Tongji University School of Medicine.

Note that full information on the approval of the study protocol must also be provided in the manuscript.

Field-specific reporting

Please select the one below that is the best fit for your research. If you are not sure, read the appropriate sections before making your selection.

[x] Life sciences [ ] Behavioural & social sciences [ ] Ecological, evolutionary & environmental sciences

For a reference copy of the document with all sections, see nature.com/documents/nr-reporting-summary-flat.pdf

Life sciences study design

All studies must disclose on these points even when the disclosure is negative.

Sample size	A sample size of 150 human colorectal tumour specimens was chosen in this study. This sample size is sufficient for significant statistical difference of clinicopathological characteristics.
Data exclusions	No.
Replication	Each experiment was repeated at least three times.
Randomization	No randomization. Groups according to clinical parameters.
Blinding	In most experiments, samples were analyzed in a blinded manner as detailed in the Methods section. Before performing Immunohistochemistry (IHC), the researchers did not know the nature of the samples.

Reporting for specific materials, systems and methods

We require information from authors about some types of materials, experimental systems and methods used in many studies. Here, indicate whether each material, system or method listed is relevant to your study. If you are not sure if a list item applies to your research, read the appropriate section before selecting a response.

Materials & experimental systems

n/a	Involved in the study	
[ ]	[x]	Antibodies
[ ]	[x]	Eukaryotic cell lines
[x]	[ ]	Palaeontology and archaeology
[ ]	[x]	Animals and other organisms
[x]	[ ]	Clinical data
[x]	[ ]	Dual use research of concern

Methods

n/a	Involved in the study	
[x]	[ ]	ChIP-seq
[x]	[ ]	Flow cytometry
[x]	[ ]	MRI-based neuroimaging

Antibodies

Antibodies used	Antibodies that recognizes PYCR1 (13108-1-AP; 1:2000 for WB, 4μg for IP, 1:100 for IF), P5CS (17719-1-AP; 1:1000 for WB), HIF-1α (20960-1-AP; 1:1000 for WB) and Tubulin (11224-1-AP; 1:5000 for WB) was purchased from Proteintech. Antibodies that recognize ELK4 (sc-166823; 1:800 for WB, 6μg for IP, 15μg for ChIP), IGF1R (sc-81464; 1:500 for WB), p-IGF1R (sc-135767; 1:500 for WB), SIRT7 (sc-365344; 1:800 for WB, 20μg for ChIP), KLK10 (sc-100551; 1:500 for WB), CNN1 (sc-58707; 1:1000 for WB), BIP (sc-13539; 1:1000

	for WB) and Lamin B (sc-365962; 1:1000 for WB) were purchased from Santa Cruz Biotechnology. Antibodies that recognize His (T505; 1:1000 for WB), AKT (phospho-ser473) (11054; 1:1000 for WB), ERK1/2 (29162; 1:1000 for WB) and ERK1/2 (phospho-Thr202/Tyr204) (12082; 1:1000 for WB) were purchased from Signalway Antibody. Antibody that recognize AKT (9272S; 1:1000 for WB) was purchased from Cell Signaling Technology. Antibodies that recognize PYCR1 (ab103314; 1:100 for IHC), Phospho-tyrosine (ab179530; 1:1000 for WB), HIF-1α (ab243860; 1:100 for IF; 1:200 for IHC) and H3K18ac (ab40888; 10μg for ChIP) were purchased from Abcam. Antibody that recognize β-actin (AC026; 1:1000 for WB) was purchased from ABclonal. Antibody that recognize Flag (F1804; 1:1000 for WB, 4μg for IP, 10μg for ChIP) was purchased from Sigma-Aldrich. Rabbit polyclonal PYCR1 pTyr135 (1:800 for WB, 1:100 for IHC, 1:50 for IF) antibody was made by Signalway Antibody.
Validation	Validations are based on the datasheets from the manufactures. All primary antibodies have been validated by Western blot using specific shRNA or over-expressing plasmid.

Eukaryotic cell lines

Policy information about cell lines and Sex and Gender in Research

Cell line source(s)	The cell lines (HCT116, SW620 and SW480) used in this study obtained from American Type Culture Collection.
Authentication	Cell-lines were authenticated by ATCC. All cell lines were authenticated by fingerprinting of short tandem repeats (STR).
Mycoplasma contamination	The cells are routinely tested for mycoplasma contamination. All cell lines tested negative for mycoplasma contamination
Commonly misidentified lines (See ICLAC register)	No.

Animals and other research organisms

Policy information about studies involving animals; ARRIVE guidelines recommended for reporting animal research, and Sex and Gender in Research

Laboratory animals	Five-week-old male nu/nu mice. The mice were allowed free access to food and water and were maintained on a 12/12 h light–dark cycle at room temperature (22-26 °C) with relative humidity around 40%.
Wild animals	The study did not involve wild animals.
Reporting on sex	No.
Field-collected samples	The study did not involve samples collected from the field.
Ethics oversight	All animal experiments conformed to the guidelines of the Institutional Animal Care and Use Committee of Tongji University.

Note that full information on the approval of the study protocol must also be provided in the manuscript.
