# Supplementary material for: IGF1R-phosphorylated PYCR1 facilitates ELK4 transcriptional activity and sustains tumor growth under hypoxia
Source: Nat Commun. 2023 Sep 30;14:6117. doi: 10.1038/s41467-023-41658-z (PMC10542766; doi:10.1038/s41467-023-41658-z)
Supplement: Supplementary file 7 — Source Data [file 41467_2023_41658_MOESM7_ESM.zip › Source Data-uncropped western blots.pdf]

Fig. 1

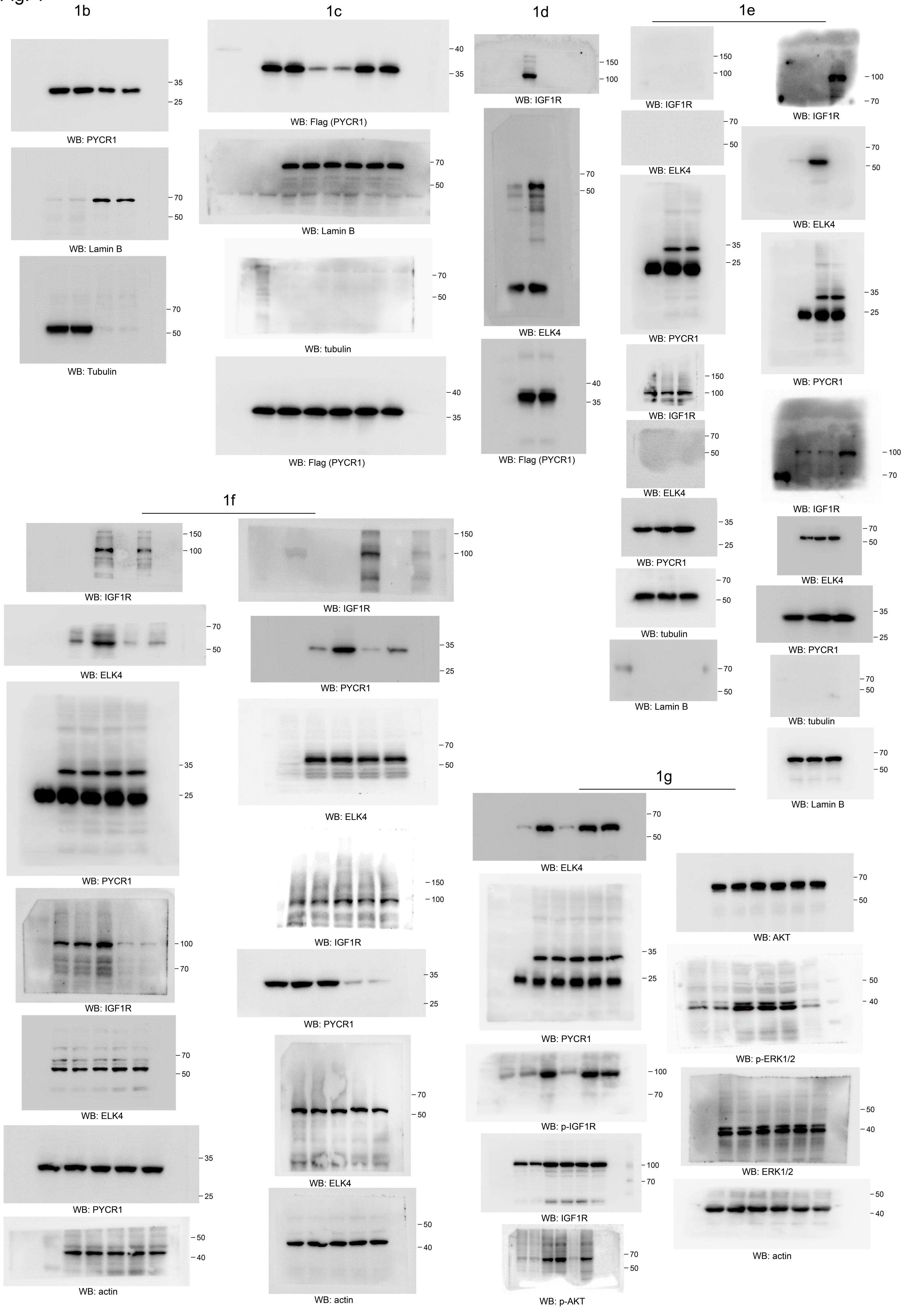

1b
WB: PYCR1
WB: Lamin B
WB: Tubulin
1c
WB: Flag (PYCR1)
WB: Lamin B
WB: tubulin
WB: Flag (PYCR1)
1d
WB: IGF1R
WB: ELK4
WB: Flag (PYCR1)
1e
WB: IGF1R
WB: ELK4
WB: PYCR1
WB: IGF1R
WB: ELK4
WB: PYCR1
WB: tubulin
WB: Lamin B
WB: IGF1R
WB: ELK4
WB: PYCR1
WB: IGF1R
WB: ELK4
WB: PYCR1
WB: tubulin
WB: Lamin B
1f
WB: IGF1R
WB: ELK4
WB: PYCR1
WB: IGF1R
WB: ELK4
WB: PYCR1
WB: actin
WB: IGF1R
WB: PYCR1
WB: ELK4
WB: IGF1R
WB: PYCR1
WB: ELK4
WB: actin
1g
WB: ELK4
WB: PYCR1
WB: p-IGF1R
WB: IGF1R
WB: p-AKT
WB: AKT
WB: p-ERK1/2
WB: ERK1/2
WB: actin

Fig. 2

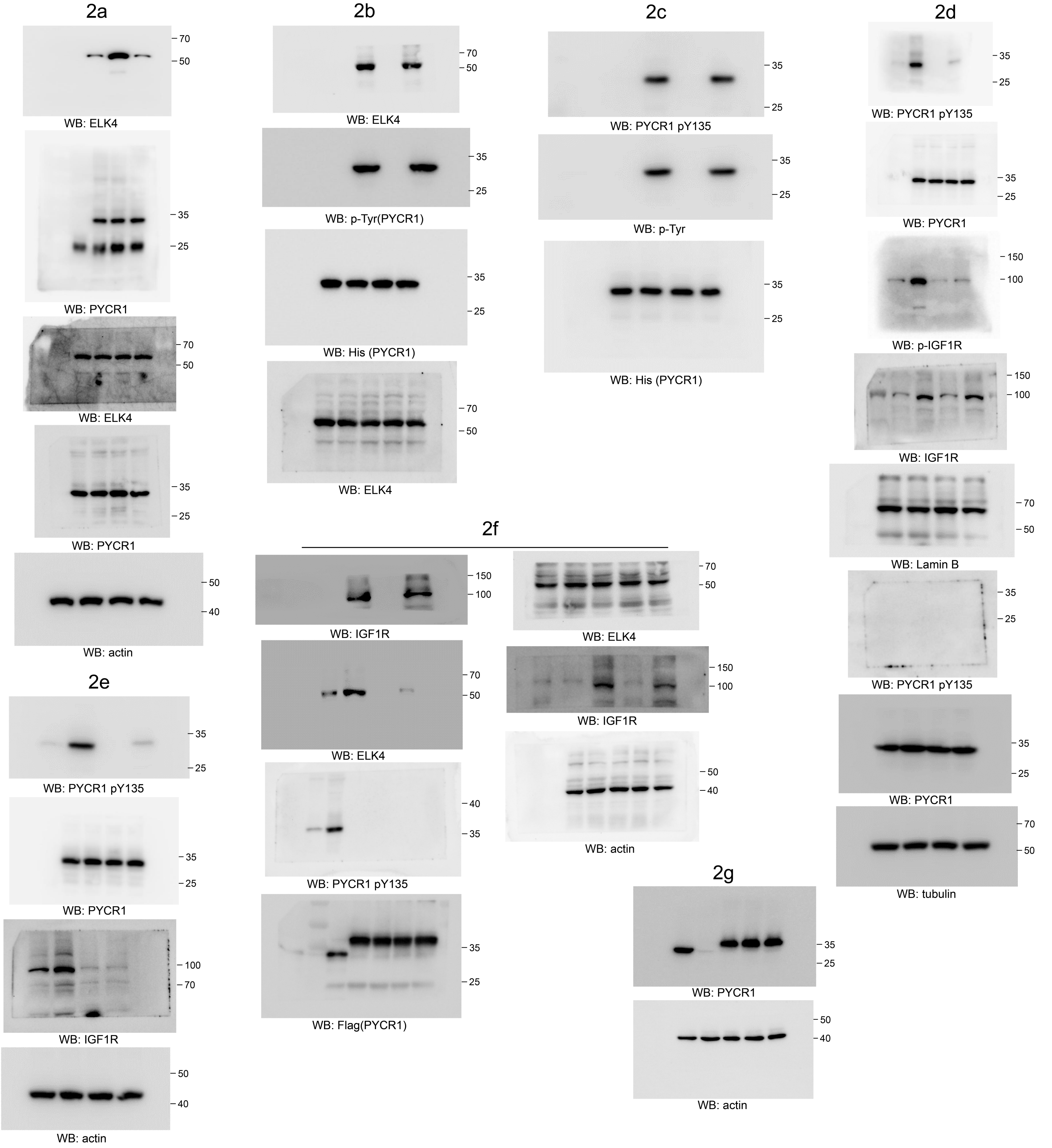
2a
WB: ELK4
WB: PYCR1
WB: ELK4
WB: PYCR1
WB: actin
2e
WB: PYCR1 pY135
WB: PYCR1
WB: IGF1R
WB: actin
2b
WB: ELK4
WB: p-Tyr(PYCR1)
WB: His (PYCR1)
WB: ELK4
2f
WB: IGF1R
WB: ELK4
WB: PYCR1 pY135
WB: Flag(PYCR1)
WB: ELK4
WB: IGF1R
WB: actin
2c
WB: PYCR1 pY135
WB: p-Tyr
WB: His (PYCR1)
2g
WB: PYCR1
WB: actin
2d
WB: PYCR1 pY135
WB: PYCR1
WB: p-IGF1R
WB: IGF1R
WB: Lamin B
WB: PYCR1 pY135
WB: PYCR1
WB: tubulin

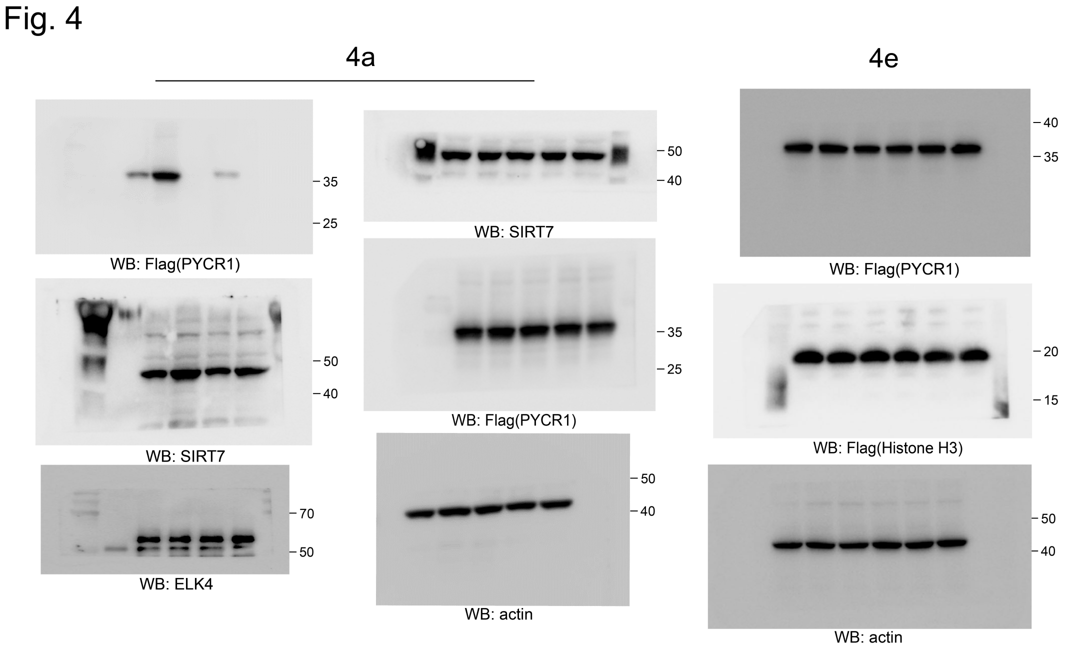
Fig. 4
4a
4e
35
25
WB: Flag(PYCR1)
50
40
WB: SIRT7
50
40
WB: SIRT7
35
25
WB: Flag(PYCR1)
70
50
WB: ELK4
50
40
WB: actin
40
35
WB: Flag(PYCR1)
20
15
WB: Flag(Histone H3)
50
40
WB: actin

Fig. 6

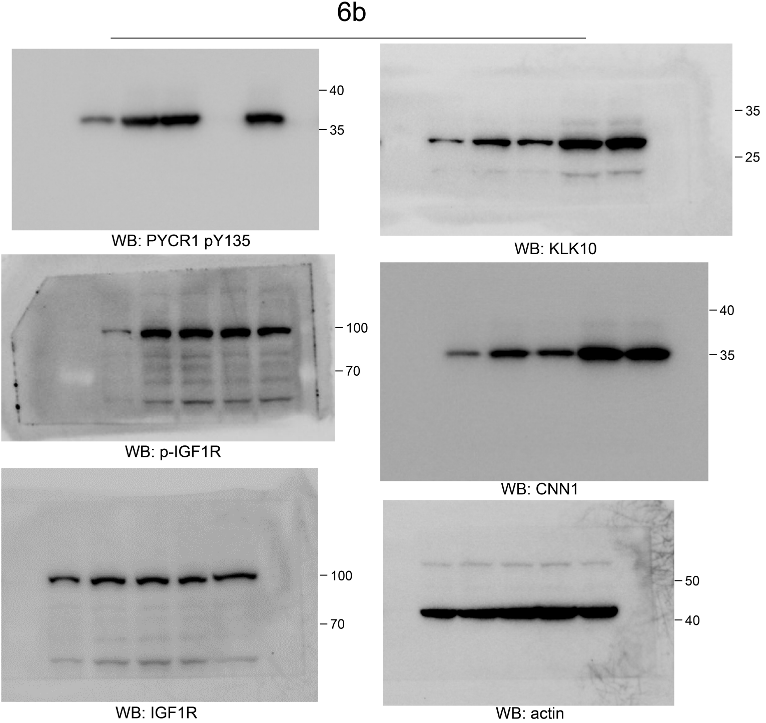
6b
40
35
WB: PYCR1 pY135
35
25
WB: KLK10
100
70
WB: p-IGF1R
40
35
WB: CNN1
100
70
WB: IGF1R
50
40
WB: actin

Supplementary Fig. 1

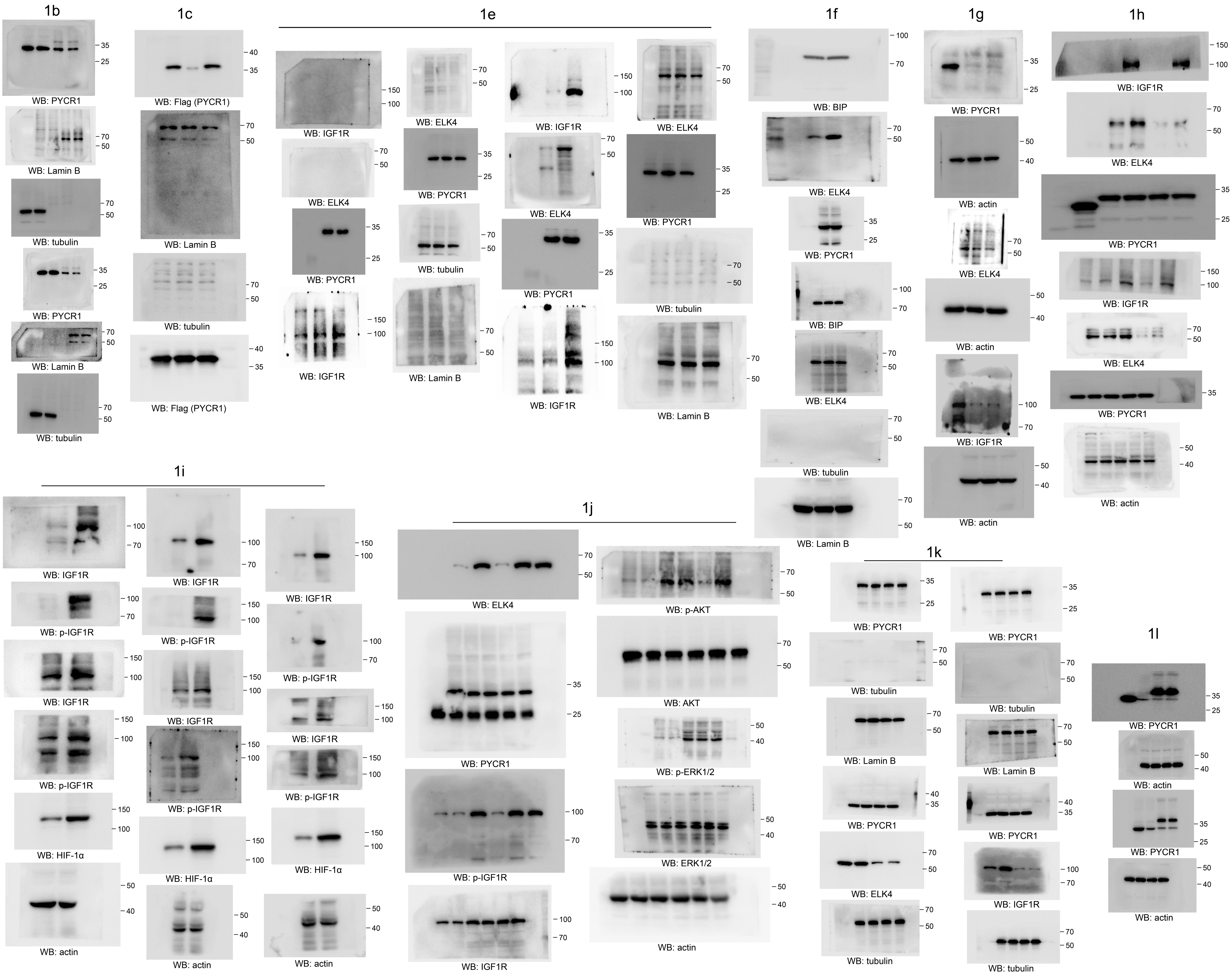

Supplenmentary Fig. 2

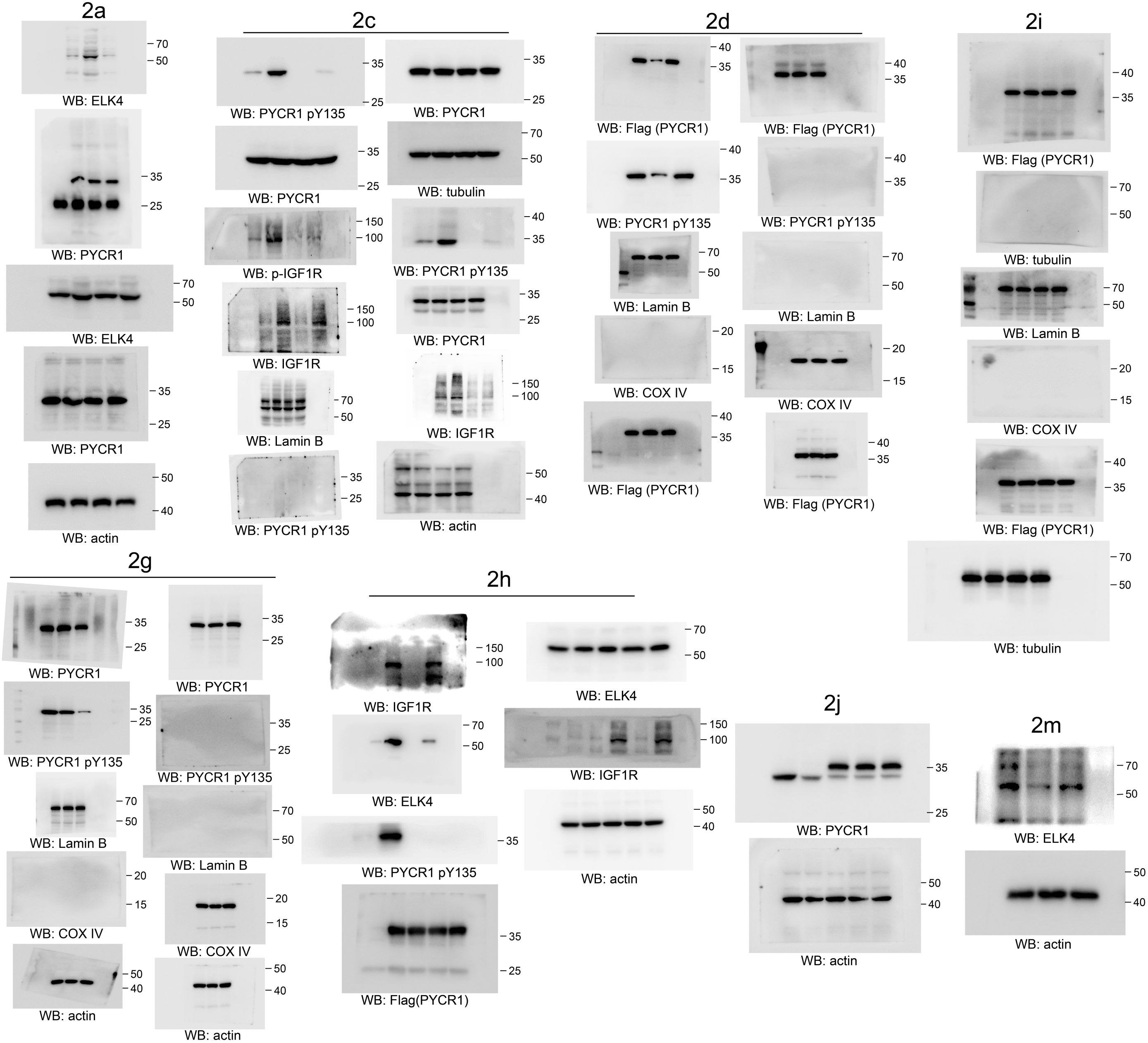

Supplementary Fig. 3

3e

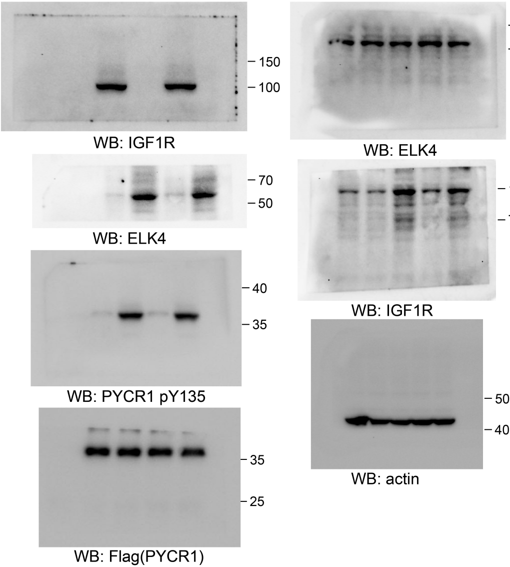

Supplementary Fig. 4

4a

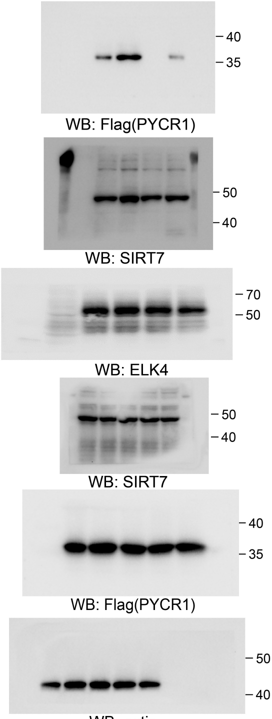

4b

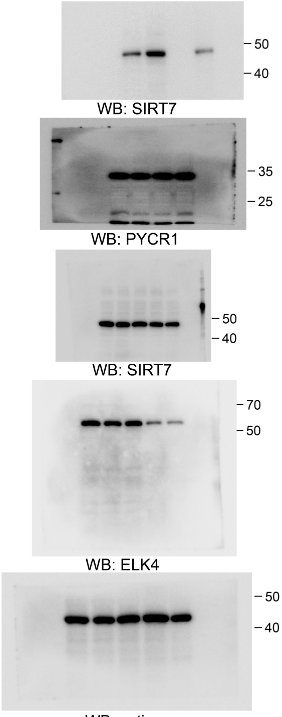

4c

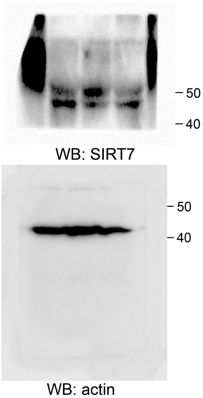

Supplementary Fig. 5

5a

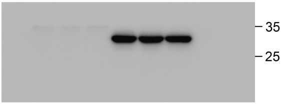

WB: HA (NMNAT1)

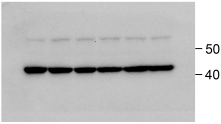

WB: actin

5e

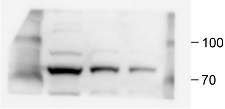

WB: P5CS

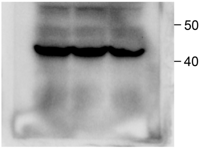

WB: actin

5h

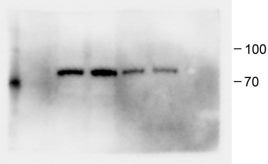

WB: P5CS

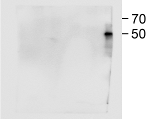

WB: tubulin

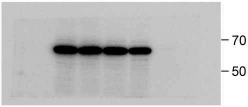

WB: Lamin B

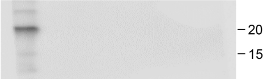

WB: COX IV

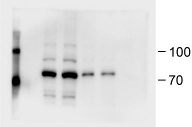

WB: P5CS

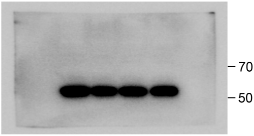

WB: tubulin

Supplementary Fig. 6

6d

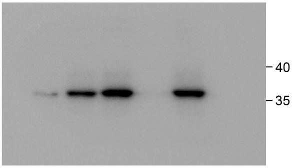

WB: PYCR1 pY135

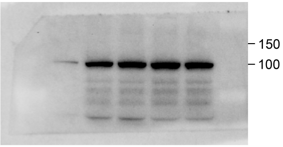

WB: p-IGF1R

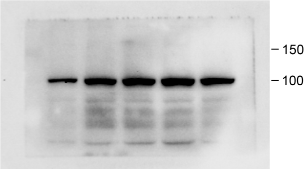

WB: IGF1R

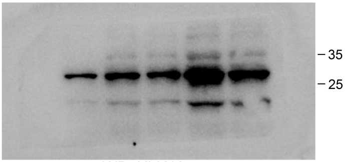

WB: KLK10

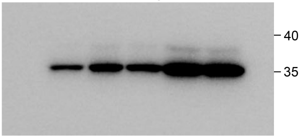

WB: CNN1

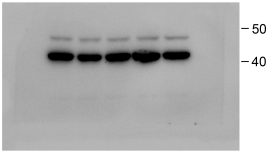

WB: actin

6e

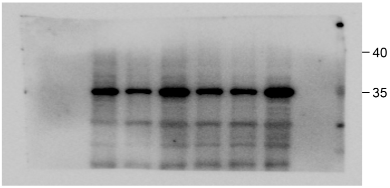

WB: PYCR1 pY135

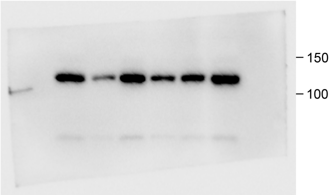

WB: HIF-1α

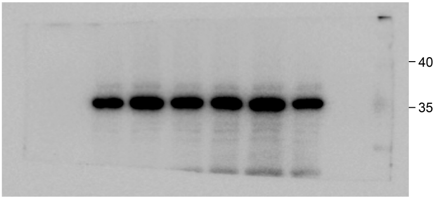

WB: PYCR1

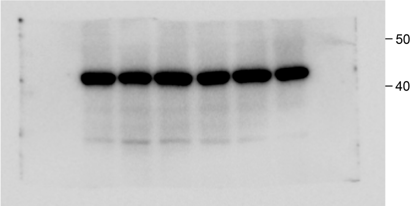

WB: actin
